# Additional Value of Pertechnetate Scintigraphy to American College of Radiology Thyroid Imaging Reporting and Data Systems and European Thyroid Imaging Reporting and Data Systems for Thyroid Nodule Classification in Euthyroid Patients

**DOI:** 10.3390/cancers16244184

**Published:** 2024-12-16

**Authors:** Lea Sollmann, Maria Eveslage, Moritz Fabian Danzer, Michael Schäfers, Barbara Heitplatz, Elke Conrad, Daniel Hescheler, Burkhard Riemann, Benjamin Noto

**Affiliations:** 1 Department of Nuclear Medicine, University of Münster and University Hospital Münster, 48149 Münster, Germany; 2 Institute of Biostatistics and Clinical Research, University of Münster, 48149 Münster, Germany; 3 West German Cancer Centre (WTZ), University Hospital Münster, 48149 Münster, Germany; 4 European Institute for Molecular Imaging, University of Münster, 48149 Münster, Germany; 5 Institute of Pathology, University Hospital of Münster, 48149 Münster, Germany; 6 Department of Radiology and Nuclear Medicine, University Hospital Magdeburg, 39120 Magdeburg, Germany; 7 Clinic for Radiology, University of Münster and University Hospital Münster, 48149 Münster, Germany

**Keywords:** thyroid cancer, thyroid nodules, TIRADS, scintigraphy, radionuclide scanning, ultrasound

## Abstract

**Background:** Thyroid nodules are common yet remain a diagnostic challenge. While ultrasound and Thyroid Imaging Reporting and Data Systems (TIRADS) are accepted as standard, the use of thyroid scintigraphy in euthyroid patients is debated. The European Association of Nuclear Medicine advocates it, whereas the American Thyroid Association and European Thyroid Association do not. However, it has not been evaluated whether scintigraphy adds value to TIRADS in a multimodal approach. Our study addresses this gap by assessing the impact of integrated pertechnetate scintigraphy on TIRADS accuracy. **Methods:** The diagnostic performance of ACR-TIRADS, EU-TIRADS, pertechnetate scintigraphy, and multimodal models were retrospectively analyzed for 322 nodules (231 benign, 91 malignant) in 208 euthyroid patients with histopathology as a reference. Generalized estimating equations were used for statistical analysis. **Results:** On scintigraphy, 210 nodules were hypofunctional, 99 isofunctional, and 13 hyperfunctional. The AUC for thyroid scintigraphy, ACR-TIRADS, and EU-TIRADS were 0.6 (95% CI: 0.55–0.66), 0.83 (95% CI: 0.78–0.88), and 0.78 (95% CI: 0.72–0.83). Integrating scintigraphy with ACR-TIRADS and EU-TIRADS slightly increased diagnostic accuracy (AUC 0.86 vs. 0.83, *p* = 0.039 and AUC 0.80 vs. 0.78, *p* = 0.008) and adjusted the malignancy probability for intermediate risk TIRADS categories, with iso- or hyperfunctioning nodules in ACR-TIRADS-TR4 or EU-TIRADS-4 showing comparable malignancy probabilities as hypofunctioning nodules in TR3 or EU-TIRADS-3, respectively. **Conclusions:** Integrating thyroid scintigraphy with ACR- or EU-TIRADS moderately improves diagnostic performance, potentially benefiting management, especially in complex cases like multinodular goiter or indeterminate FNA. Further research is warranted to validate these findings and explore their clinical implications.

## Simple Summary

Thyroid nodules are a frequent finding yet remain a diagnostic challenge. While ultrasound and Thyroid Imaging Reporting and Data Systems (TIRADS) are widely accepted, the role of thyroid scintigraphy in euthyroid patients is debated. The European Association of Nuclear Medicine advocates its use, whereas the American Thyroid Association (ATA) and European Thyroid Association (ETA) do not include scintigraphy in their diagnostic algorithms. However, no study has systematically quantified whether scintigraphy adds diagnostic value to TIRADS in a multimodal approach. Our study addresses this gap, demonstrating a moderate improvement in diagnostic performance when incorporating scintigraphy into both ACR-TIRADS and EU-TIRADS, providing more accurate risk assessment, particularly in TIRADS categories with intermediate malignancy risk. This combined approach may benefit complex cases, such as multinodular goiter or indeterminate fine-needle aspiration results. Further research to determine if pertechnetate scintigraphy benefits the evaluation of thyroid nodules in selected cases of euthyroid patients seems warranted.

## 1. Introduction

Thyroid nodules are a very common, often incidental clinical finding, detectable on high-resolution ultrasound in 25% to 75% of the general population [1,2]. While the majority of these nodules prove to be benign, up to 15% exhibit malignant characteristics, with thyroid cancer representing the most common endocrine malignancy [3,4,5].

Ultrasound is the primary imaging modality for the work-up of thyroid nodules in euthyroid patients. In recent years, several Thyroid Imaging Reporting and Data System (TIRADS), based on B-mode ultrasound characteristics, have been developed to this end [3,6,7,8]. Another imaging modality with a long history of use in thyroid evaluation is radionuclide scanning, mainly with [^*99m*^Tc]Tc-pertechnetate. In contrast to ultrasound, thyroid scintigraphy primarily gives insight into the functional status. However, the use of thyroid scintigraphy for evaluating thyroid nodules in euthyroid patients is controversial.

The current German guidelines recommend the scintigraphic evaluation of every thyroid nodule larger than 1 cm [9]. The European Association of Nuclear Medicine recommends using thyroid scintigraphy to preselect only “cold” nodules for fine needle aspiration cytology (FNA), claiming that this strategy can greatly reduce the number of thyroid surgical procedures for benign entities, especially in populations with a high prevalence of iodine deficiency [10,11]. This implies that malignancy can be excluded with a high degree of certainty in isofunctional or hyperfunctional nodules. More so, hyperfunctioning nodules are considered to be virtually exclusively benign [12]. The American Thyroid Association (ATA) mentions the use of thyroid scintigraphy as a possibility for FNA-preselection in multinodular goiter but does not provide further details [3]. The European Thyroid Association mentions that thyroid scintigraphy may show hyperfunctioning nodules even in patients with normal TSH values and claims that hyperfunctioning nodules are rarely malignant. However, thyroid scintigraphy is not included in the proposed algorithm for the workup of thyroid nodules in euthyroid patients [13]. Finally, the British NICE-Guidelines recommend against the use of radionuclide scanning, basing their decision on insufficient and weak evidence concerning the use of thyroid scintigraphy in the evaluation of thyroid nodules in euthyroid patients [14].

Additionally, even the guidelines and societies that favor the use of thyroid scintigraphy for nodule evaluation do not elaborate on how to interpret the results of radionuclide scanning in conjunction with ultrasound in a multimodal approach.

Therefore, the aim of this study was to investigate whether thyroid scintigraphy adds diagnostic value to ACR- and EU-TIRADS in a multimodal approach.

## 2. Materials and Methods

### 2.1. Patients and Data Recruitment

Two separately recruited patient groups were retrospectively evaluated for this analysis. Inclusion criteria were the availability of preoperative thyroid ultrasound, pertechnetate scintigraphy, laboratory results, and pathology reports following thyroidectomy. Furthermore, only nodules ≥ 1 cm on ultrasound were included. Patients with Grave’s disease or hyperthyroidism from other causes were excluded. The first group (Group 1) consisted of all patients presenting for thyroid cancer follow-up care at a tertiary referral hospital (*n* = 100) in 2022 and 2023, with nodal work-up performed at an outside facility. After informed consent was obtained, initial imaging was requested from external nuclear medicine specialists who performed the initial evaluation of the thyroid. The second group of patients (Group 2) comprised euthyroid patients who presented for thyroid nodule work-up between 2011 and 2023 in the same department (*n* = 108). It should be noted that the original data set could be analyzed for each sonographic evaluation, as these were either stored in our system or the external facilites were able to provide us with their originals.

Eligible subjects who met inclusion criteria were identified. The complete preoperative data and pathology reports of the thyroidectomy specimens were available for 245 patients. For the evaluation of the diagnostic value of thyroid scintigraphy, TIRADS, and their combination, 21 patients were excluded due to hyperthyroidism, a further 5 because the pathology report could not be properly correlated with the nodules identified on imaging, 5 because of the insufficient image quality of the preoperative ultrasound or scintigraphy images, and 6 because the nodules were smaller than 1 cm. Preoperative imaging dated from February 2011 to July 2023.

The study protocol was approved by the ethics committee of the University of Münster (2022-428-f-S) and performed in accordance with the ethical standards as laid down in the 1964 Declaration of Helsinki and its later amendments.

### 2.2. Imaging and Image Analysis

In the 108 patients who had their thyroid nodule work-up in our department (Group 2), ultrasound of the thyroid gland was performed using either a Philips iU22 (Philips Healthcare, Eindhoven, The Netherlands), a Siemens Acuson NX3 Elite (Siemens Healthineers, Erlangen, Germany), or a Siemens Acuson S1000 (Siemens Healthineers, Erlangen, Germany) with a linear probe and a frequency of 5–10 MHz. Thyroid scintigraphy was acquired according to German guidelines 10–25 min post-injection of 70 MBq [^*99m*^Tc]Tc-pertechnetate with a MIE Scintron (MiE Germany, Seth, Germany (LEHR collimation, matrix 128 × 128; acquisition time 10 min). One hundred patients (group 1) had their initial thyroid nodule work-up in various external institutions. Accordingly, different ultrasound and scintigraphy systems were used.

Ultrasound images were retrospectively classified according to the ACR-TIRADS [6] system by two experienced nuclear medicine specialists in a blinded fashion. The composition of nodules; echogenicity; shape; margin; and presence of echogenic foci were noted. On this basis, the corresponding EU-TIRADS categories of nodules were calculated. Nodule functional status was retrospectively assessed on preoperative [^*99m*^Tc]Tc-pertechnetate thyroid scintigraphy scans as either hypofunctional (below the level of the surrounding thyroid tissue), isofunctional (at the level of the surrounding thyroid tissue), or hyperfunctional (above the level of the surrounding thyroid tissue) [15]. Only nodules that could be unequivocally correlated between ultrasound, scintigraphy, and pathology reports were included for further analysis.

### 2.3. Statistical Analyses

The distribution of quantitative parameters is summarized using the median and the interquartile range [lower–upper quartile]. The distribution of categorical variables is presented using absolute and relative frequencies, while 95% confidence intervals for proportions were calculated using the Wilson method. The association of categorical variables is visualized using bar charts and mosaic plots.

Multiple thyroid nodules per patient could be measured. To account for the resulting clustered nature of the data, further statistical analyses were based on generalized estimating equations (GEE) [16,17]. Measures of diagnostic performance and corresponding confidence intervals were calculated on a per nodule basis based on parameter estimates as well as the robust variance-covariance matrix resulting from the GEE approach [18]. The diagnostic performance of scintigraphy, the ACR-TIRADS, as well as the EU-TIRADS was quantified in terms of sensitivity, specificity, diagnostic odds ratio (DOR), as well as positive and negative diagnostic likelihood ratio (DLR+ and DLR−) [19]. The probability of malignancy was estimated depending on the result of the scintigraphy and the ACR-TIRADS or the EU-TIRADS. Confidence intervals for sensitivity, specificity, and the probability of malignancy were calculated using the back-transformation approach. Confidence intervals for DLR+ and DLR− were calculated by applying the delta method to the log-transformed estimator followed by a back-transformation.

The diagnostic quality of different parameters was compared based on the area under the ROC curve (AUC) [20]. ROC curves facilitate the comparison of diagnostic methods, irrespective of the prevalence or the decision threshold. As sensitivity and specificity are independent of prevalence, the AUC is also independent of prevalence [19,21].

All analyses are intended as exploratory, as no adjustment for multiple testing was applied. The analyses were performed using R version 4.3.2 [22] and SAS ^®^ software, version 9.4, for Windows (SAS Institute, Cary, NC, USA).

## 3. Results

### 3.1. Patient and Nodule Characteristics

Two hundred and eight patients met the inclusion criteria, with 68.9% being female and a median age of 47 years [37.3–58.0]. Chronic lymphocytic thyroiditis was identified in 28 patients based on pathology reports. Of the included patients, 129 were diagnosed with thyroid cancer, including papillary (98 cases), follicular (19 cases), anaplastic (1 case), medullary (4 cases), oncocytic (5 cases), and poorly differentiated (2 cases). Seventy-nine patients exhibited solely benign histological findings. The median number of nodules per patient was 1, with a maximum of 6. The median preoperative TSH level was 1.37 µU/mL [0.71–2.04 µU/mL].

The correlation of imaging and pathology reports was possible for 322 nodules (231 benign, 91 malignant). Note that, although 129 patients were diagnosed with thyroid cancer, only 91 malignant nodules were included in this analysis. This discrepancy arose because 44 patients with papillary microcarcinoma were included due to the presence of benign nodules larger than 1 cm, while microcarinomas were not included due to their size being less than 1 cm. One instance of non-invasive follicular thyroid neoplasm with papillary-like nuclear features (NIFTP) was classified as benign. Benign nodules had a median size of 20 mm [15–31 mm], and malignant nodules had a median size of 20 mm [15–32 mm].

**Figure 1:**
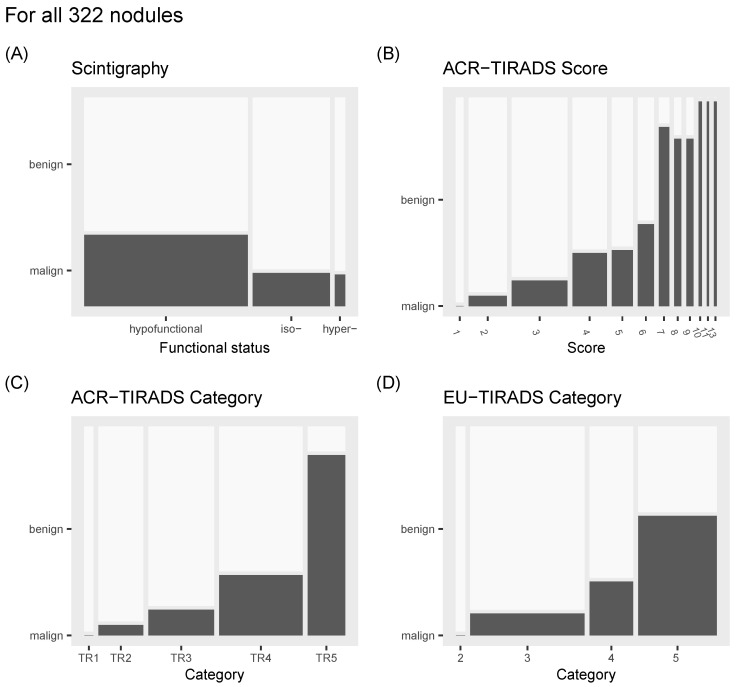
Mosaic plot illustrating the relationship between nodule status and (**A**) functional status assessed by scintigraphy, (**B**) ACR-TIRADS score, (**C**) ACR-TIRADS category, and (**D**) EU-TIRADS category. The width of each vertical segment represents the proportion of nodules that are hypofunctional, isofunctional, or hyperfunctional on thyroid scintigraphy (**A**), and the proportion of nodules in each ACR-TIRADS score (**B**), ACR-TIRADS category (**C**), and EU-TIRADS category (**D**). The height of each colored section corresponds to the proportion of malignant nodules, with full height representing 100%.

On thyroid scintigraphy, 210 (65.22%) nodules were hypofunctional, 99 (30.75%) were isofunctional, and 13 (4.04%) were hyperfunctional, while 137, 83, and 11 out of 231 benign nodules and 73, 16, and 2 out of 91 malignant nodules were hypo-, iso-, or hyperfunctional, respectively. The malignancy rate of hypofunctional nodules was 34.8% (73/210), compared to 16.2% (16/99) for isofunctioning nodules and 15.4% (2/13) for hyperfunctioning nodules (see Figure 1).

The distribution of benign and malignant thyroid nodules across ACR-TIRADS scores/categories and EU-TIRADS categories, as well as the nodules’ distribution among the categories and scores, is illustrated in Figure 1.

### 3.2. Diagnostic Performance of Thyroid Scintigraphy, ACR-TIRADS, and EU-TIRADS

As demonstrated, the malignancy rates for iso- and hyperfunctioning nodules were comparable—16.16% and 15.38%, respectively—while hypofunctioning nodules exhibited a higher malignancy rate of 34.76%. Based on these distinctions in malignancy rates, we analyzed the performance of thyroid scintigraphy by classifying it into two categories: hypofunctioning nodules and a combined group of isofunctioning and hyperfunctioning nodules. Our analysis revealed a sensitivity of 80% (95% CI: 0.71–0.88) and a specificity of 39% (95% CI: 0.32–0.47), with a positive likelihood ratio of 1.32 (95% CI: 1.13–1.56), a negative likelihood ratio of 0.50 (95% CI: 0.31–0.81), and a diagnostic odds ratio of 2.65 (95% CI: 1.41–4.98) (see Table 1).

Receiver operating characteristic (ROC) analysis revealed virtually identical areas under the curve (AUC) for thyroid scintigraphy when categorized as a two- or three-level variable. Specifically, the AUC was 0.60 (95% CI: 0.55–0.66) on the two-level basis and 0.60 (95% CI: 0.55–0.66) on the three-level basis. Notably, the AUC of thyroid scintigraphy, whether categorized as a two- or three-level variable, was significantly lower than that of ACR-TIRADS at both the score level (0.60 versus 0.84, *p* < 0.001) and the category level (0.60 versus 0.83, *p* < 0.001), as well as compared to EU-TIRADS (0.60 versus 0.78, *p* < 0.001).

The diagnostic performance characteristics of ACR-TIRADS and EU-TIRADS for various cutoffs are detailed in Table 1. The AUC of ACR-TIRADS outperformed that of EU-TIRADS, both at the category and score levels (0.83 and 0.84 vs. 0.78, respectively, both *p* < 0.001).

### 3.3. Diagnostic Value of Combined TIRADS and Pertechnetate Scintigraphy

**Figure 2:**
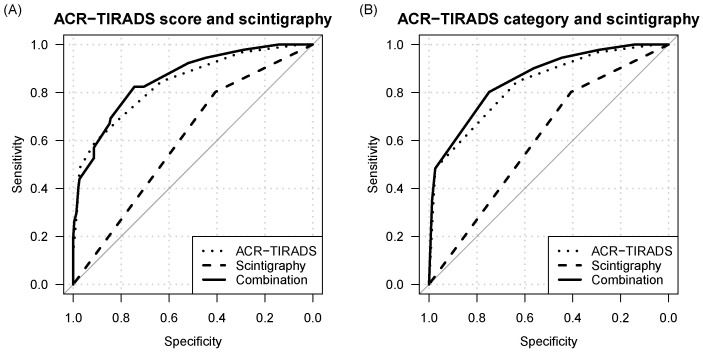
ROC curves for: (**A**) thyroid scintigraphy (dashed line, AUC: 0.6), ACR-TIRADS score (dotted line, AUC: 0.84), and the combined model (solid line, AUC: 0.86). (**B**) Thyroid scintigraphy (dashed line, AUC: 0.6), ACR-TIRADS categories (dotted line; AUC: 0.83), and the combined model (solid line, AUC: 0.86).

**Figure 3:**
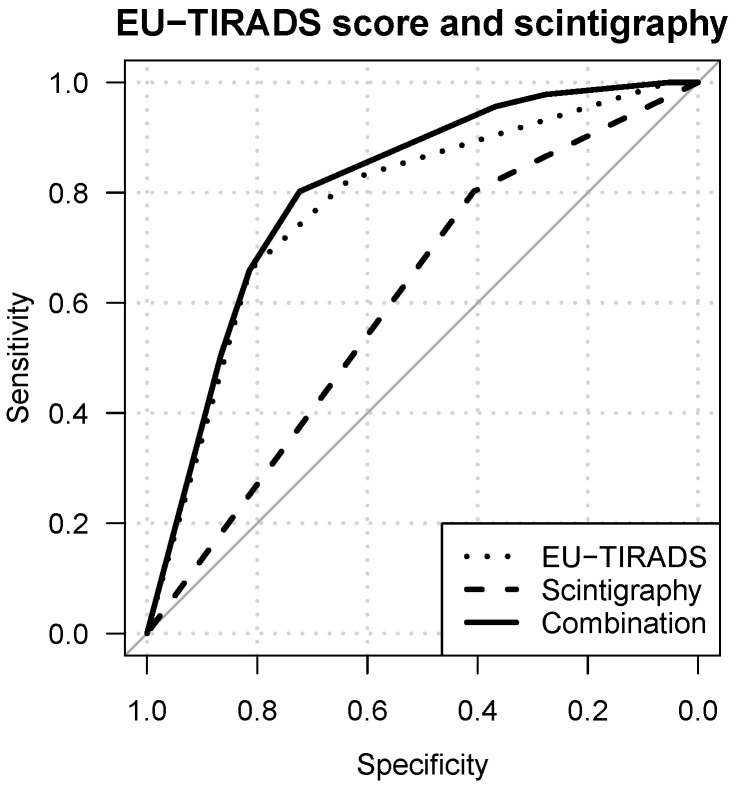
ROC curves for: thyroid scintigraphy (dashed line, AUC: 0.6), EU-TIRADS (dotted line; 0.78), and the combined model (solid line, AUC: 0.80).

To evaluate whether incorporating radionuclide scanning alongside ACR- or EU-TIRADS enhances diagnostic efficacy, we conducted a comparative analysis of the area under the curve (AUC) between standalone TIRADS and multimodal TIRADS/scintigraphy models. One of the evaluated models combined ACR-TIRADS at the category level and scintigraphy as a two-level variable (hypofunctional vs. isofunctional and hyperfunctional). The integrated model demonstrated a superior AUC of 0.86 in contrast to 0.83 for ACR-TIRADS alone (*p* = 0.039). Similarly, the composite model pairing EU-TIRADS with scintigraphy as a two-level variable exhibited enhanced performance over EU-TIRADS alone in terms of AUC (0.80 versus 0.78, *p* = 0.008). Although the model combining ACR-TIRADS on a score level with scintigraphy showed a higher AUC than ACR-TIRADS on a score level alone, the difference did not reach statistical significance (0.86 vs. 0.84, *p* = 0.064). Further details regarding performance and differences between other tested models can be found in Table 2. Receiver operating characteristic (ROC) curves for standalone and combined models are shown in Figure 2 and Figure 3.

**Figure 4:**
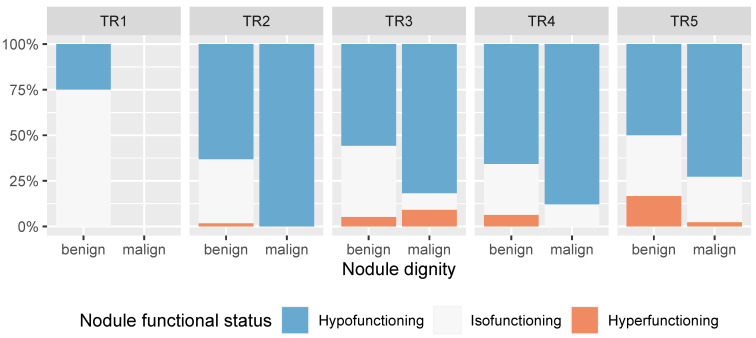
Barplot showing the relation of nodule dignity and functional status as assesed by scintigraphy, stratified by ACR-TIRADS category (*n* = 322 nodules in 208 patients). The proportion of hypofunctioning nodules is higher in malignant nodules in all ACR-TIRADS categories. Note that there are malignant hyperfunctional nodules.

**Figure 5:**
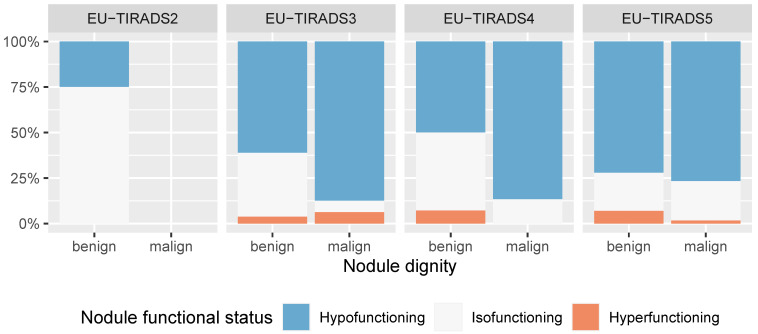
Barplot showing the relation of nodule dignity and functional status as assessed by scintigraphy, stratified by EU-TIRADS category (*n* = 322 nodules in 208 patients).

As shown in Figure 4, malignant nodules were more frequently hypofunctional than benign nodules across all ACR-TIRADS categories except TR1, which had no malignant nodules. Similarly, malignant nodules were more frequently hypofunctional than benign nodules across all EU-TIRADS categories (except EU-TIRADS orange2, which had no malignant nodules), underscoring the potential of radionuclide scanning to further stratify nodules based on the probability of malignancy (Figure 5). 

**Figure 6:**
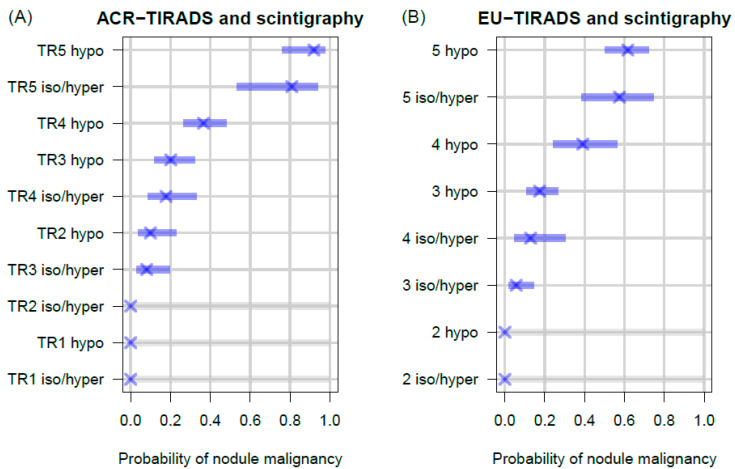
Estimated probability for nodule malignancy (purple cross) and corresponding 95% confidence intervals (bars, *n* = 322. (**A**) Combinations of ACR-TIRADS categories and thyroid scintigraphy. Note that the probability of malignancy for a hyper- or isofunctioning nodule in the category TR4 is comparable to a hypofunctional nodule in the category TR3. The probability of malignancy for a iso-/or hyperfunctioning nodule in the category TR3 is comparable to a hypofunctioning nodule in the category TR2 (Confidence intervals for hyper- or isofunctioning nodules in categories TR1 and TR2 could not be estimated; hence, their error bars are greyed out for visual distinction). (**B**) Combinations of EU-TIRADS categories and thyroid scintigraphy. Note that the probability of malignancy for a hyper- or isofunctioning nodule in the category EU-TIRADS 4 is comparable to a hypofunctional nodule in the category EU-TIRADS 3 (confidence intervals cannot be estimated for category EU-TIRADS 2; hence, their error bars are greyed out for visual distinction).

Indeed, integrating scintigraphy into the two TIRADS schemes led to a reordering of the estimated probability for malignancy across TIRADS categories (Figure 6). Notably, for ACR-TIRADS, the probability of malignancy for a hyper- or isofunctioning nodule in the TR4 category was comparable to that of a hypofunctional nodule in the TR3 category. Similarly, the probability of malignancy for an isofunctioning or hyperfunctioning nodule in the TR3 category was comparable to that of a hypofunctional nodule in the TR2 category. For EU-TIRADS, the probability of malignancy for a hyper- or isofunctioning nodule in the EU-TIRADS 4 category was comparable to that of a hypofunctional nodule in the EU-TIRADS 3 category (Figure 6).

## 4. Discussion

This retrospective study assessed the diagnostic efficacy of [^*99m*^Tc]Tc-pertechnetate radionuclide scanning and two ultrasound-based Thyroid Imaging Reporting and Data Systems (TIRADS)—the American College of Radiology (ACR-TIRADS) and the European (EU-TIRADS)—for classifying thyroid nodules in euthyroid patients. Our investigation evaluated these methodologies both independently and within a multimodal framework, demonstrating that the integration of pertechnetate thyroid scintigraphy with ACR-TIRADS or EU-TIRADS enhances predictive accuracy beyond the capabilities of either TIRADS alone moderately.

The area under the receiver operating characteristic curve (AUC) was used to quantify and compare the diagnostic accuracy of different methods. The AUC calculated in our study can be interpreted as the probability that a randomly selected malignant nodule has a test result indicating greater suspicion than a randomly chosen non-malignant nodule [19,23]. Unlike both the negative and positive predictive values, the AUC assesses the diagnostic effectiveness of a test independently of disease prevalence.

Concerning the diagnostic performance of the investigated TIRADS, our findings revealed an AUC of 0.83 (95% CI: 0.78–0.88) for ACR-TIRADS. This aligns with the AUC ranges of 0.78 to 0.88 reported in prior studies [24,25,26,27,28]. For EU-TIRADS, we observed an AUC of 0.78 (95% CI: 0.72–0.83), consistent with previously reported ranges of 0.73 to 0.80 [24,25,26,28]. Comparatively, ACR-TIRADS outperformed EU-TIRADS in terms of AUC, with a statistically notable margin (0.83 vs. 0.78, *p* < 0.001), a finding echoed by Zhe Jin et al. in their analysis of 3438 thyroid nodules, primarily evaluated against surgical outcomes and histopathology [29]. Research by Sun Huh et al. and Yi-Xin Shi et al. similarly identified an advantage in AUC for ACR-TIRADS over EU-TIRADS, a distinction not observed in Seifert et al.’s study [24,30,31]. Unlike many previous studies, ours employed histopathological examination of each thyroid nodule as the reference standard, in contrast to relying on follow-up evaluations or fine-needle aspiration (FNA) for validation, providing a robust basis for comparing the diagnostic value of these methodologies.

The diagnostic performance of thyroid scintigraphy was limited compared to both ACR-TIRADS and EU-TIRADS, with an AUC of 0.60 versus 0.83 and 0.78, respectively (both *p* < 0.001). To enable comparisons with prior studies evaluating the diagnostic capacity of thyroid scintigraphy, which did not report AUC values, we conducted supplementary analyses of sensitivity and specificity. For these analyses, thyroid scintigraphy outcomes were grouped into two categories: hypofunctioning nodules in one group and iso- or hyperfunctioning nodules in the other, based on their similar malignancy rates. We observed a sensitivity of 80% (95%CI: 0.71–0.88) and a specificity of 39% (95%CI: 0.32–0.47), reflecting a pattern of relatively high sensitivity but modest specificity, aligning with existing literature. In close agreement with our results, Jones et al. reported a sensitivity of 82% and a specificity of 34% in their study of 175 euthyroid patients [32]. Similarly, Paneerselvan et al. observed a sensitivity of 60% and a specificity of 13% in a cohort of 109 euthyroid patients [33]. The studies of Kountakis et al. (*n* = 243) and Lumachi et al. (*n* = 496) reported higher sensitivities for scintigraphy of 0.91 and 0.95, respectively, but lower specificities of 0.19 and 0.21 [34,35]. Of note, TSH-values are were not reported in those two studies, which may limit the comparability of results [34,35].

Hyperfunctioning nodules are widely regarded as almost exclusively benign [10]. However, in our study, 2 of 13 hyperfunctioning nodules were found to be malignant (15.4%, 95% CI: 4.3–42.2%). While our study was not designed to assess the prevalence of malignancy in hyperfunctioning nodules, the observed proportion of malignancies is notable if hyperfunctional nodules were indeed virtually always benign. This assumption is predominantly founded upon research conducted during the nascent stages of radioiodine diagnostics and therapy [36]. Subsequent research has reported higher malignancy rates in hyperfunctioning nodules, more in line with our findings. For example, Ashcraft et al. (1981) identified malignancy in 4% (10/283) of nodules deemed hyperfunctional on radioiodine scans, and up to 29% (6/21) on [^*99m*^Tc]Tc-pertechnetate scans [37]. Ikekubo et al. (1989) identified seven carcinomas in 16 resected hot nodules, and more recently, Dirikoc et al. (2015) reported that among 227 toxic and 31 nontoxic autonomous nodules operated on between 2008 and 2014, 20 (8.8%) and 2 (6.5%), respectively, were malignant [38,39]. Given the contrast between these findings and the prevailing assumption in the scientific literature that hyperfunctional nodules are almost exclusively benign, further research into the frequency of malignancy in hyperfunctioning nodules seems warranted.

Beyond comparing the individual diagnostic values of ultrasound-based TIRADS and thyroid scintigraphy, this study introduces an innovative perspective by systematically exploring the added diagnostic value of thyroid scintigraphy to TIRADS in a multimodal approach. Our findings suggest that integrated models combining radionuclide scanning with TIRADS improve diagnostic accuracy compared to TIRADS alone. Particularly, the integration of thyroid scintigraphy adjusted the estimated malignancy probability among TIRADS categories with intermediate risk. Comparable malignancy probabilities were observed between iso- or hyperfunctioning nodules in ACR-TIRADS TR4 and hypofunctioning nodules in TR3. Similarly, comparable malignancy probabilities were observed between the iso- and hyperfunctioning nodules in EU-TIRADS 4 and the hypofunctional nodule in the category EU-TIRADS 3, albeit with broad confidence intervals. This novel insight, pending validation by future research, holds potential clinical implications. In a study investigating 1257 nodules in 566 euthyroid patients presenting for thyroid nodule work-up, 54% of nodules were classified as TR3 or TR4 according to ACR-TIRADS and 75% as EU-TIRADS 3 or 4 according to EU-TIRADS [15]. Hence, the adjustment of estimated malignancy probability among TIRADS categories with intermediate risk, achieved by integrating thyroid scintigarphy, might have impacted a significant proportion of cases in this population. Our findings might be especially relevant in the management of multinodular goiter, potentially reducing the number of nodules necessitating fine-needle aspiration cytology (FNA). Furthermore, incorporating thyroid scintigraphy as a complementary diagnostic modality could be beneficial in up to 30% of cases where fine-needle aspiration cytology (FNA) yields indeterminate results [40]. However, the incremental diagnostic value of scintigraphy to TIRADS found in our study was only modest. Therefore, further research is essential to validate these findings and determine whether this moderate enhancement in diagnostic performance translates into meaningful clinical benefits.

Our study is subject to several limitations. Firstly, its retrospective design, which merges two distinct patient groups—Group 1, consisting of patients evaluated for thyroid nodules in our department with subsequent histopathology results, and Group 2, comprising patients undergoing thyroid cancer follow-up with available preoperative imaging—may have introduced bias. The prevalence of malignant nodules in our study population is high. Nevertheless, the comparison of the diagnostic potential of different methods remains valid as the AUC is independent of the prevalence [19,21]. Also, we consider the type of nodules a representative sample of malignant and non-malignant nodules. Therefore, our findings are transferable to patients undergoing initial nodule evaluation, where a lower prevalence of malignancy is typically expected [41]. Furthermore, approximately half of the patients underwent preoperative imaging at various institutions outside our department, leading to potential variability in imaging quality and execution. Also, all thyroid scans included in this study were conducted using [^*99m*^Tc]Tc-pertechnetate. [^*99m*^Tc]Tc-pertechnetate is taken up into thyrocytes but not organified. Previous studies have shown that about 5% of nodules hyperfunctional in 99mTc-pertechnetate are so-called trapping only nodules, i.e., they are cold in ^123^I scintigraphy [15,42]. Another limitation is that histopathological analyses were not revisited for this study, and some of the utilized reports precede the 2016 introduction of the “non-invasive follicular thyroid neoplasm with papillary-like nuclear features” (NIFTP) [43]. Consequently, certain nodules identified as malignant due to the diagnosis of the follicular variant of papillary thyroid carcinoma might actually correspond to NIFTP, which is regarded as benign in our analysis.

## 5. Conclusions

In conclusion, our study demonstrates an enhancement of diagnostic performance achieved by integrating thyroid scintigraphy with ACR- and EU-TIRADS for classifying thyroid nodules in euthyroid patients. Such a multimodal approach could improve risk stratification and management decisions, particularly in complex scenarios like multinodular goiter or with indeterminate FNA. Further research is warranted to validate these findings and explore their clinical implications.

## Figures and Tables

**Table 1:** Diagnostic performance metrics of scintigraphy, ACR-TIRADS, and EU-TIRADS using different cutoffs. Analyses based on 322 nodules in 208 patients. Numbers in brackets are 95% confidence intervals. * AUC for scintigraphy with the two levels “cold” and “indeterminate” or “warm”. AUC for ACR-TIRADS using scores. ** AUC for scintigraphy with the three levels “cold”, “indeterminate”, and “warm”. AUC for ACR-TIRADS using categories. ^†^ All 12 nodules classified as ACR-TIRADS TR1 or EU-TIRADS 2 were benign. Hence, using ACR-TIRADS ≥ 2 or EU-TIRADS ≥ 3 as a cutoff, the sensitivity was 100%, and the specificity 0.05%. For this combination, 95% confidence intervals and diagnostic likelihood ratios are not computable with the GEE-Model.

	Scintigraphy	ACR-TIRADS ≥ 2	ACR-TIRADS ≥ 3	ACR-TIRADS ≥ 4	ACR-TIRADS = 5	EU-TIRADS ≥ 3	EU-TIRADS ≥ 4	EU-TIRADS = 5
**Sensitivity**	0.80	1 ^†^	0.97	0.85	0.48	1 ^†^	0.83	0.67
	(0.71–0.88)		(0.90–0.99)	(0.76–0.91)	(0.38–0.59)		(0.74–0.90)	(0.56–0.76)
**Specificity**	0.39	0.05 ^†^	0.29	0.62	0.97	0.05 ^†^	0.62	0.81
	(0.32–0.47)		(0.23–0.36)	(0.55–0.69)	(0.94–0.99)		(0.54–0.68)	(0.74–0.86)
**DLR+**	1.32	-	1.36	2.26	18.56	-	2.17	3.46
	(1.13–1.56)		(1.23–1.50)	(1.84–2.77)	(8.23–41.82)		(1.77–2.67)	(2.52–4.77)
**DLR−**	0.50	-	0.11	0.24	0.53	-	0.27	0.41
	(0.31–0.81)		(0.04–0.36)	(0.14–0.40)	(0.43–0.65)		(0.17–0.43)	(0.30–0.56)
**DOR**	2.65	-	12.08	9.44	35.04	-	8.01	8.48
	(1.41–4.98)		(3.55–41.10)	(4.90–18.19)	(14.11–86.98)		(4.31–14.91)	(4.82–14.92)
AUC *	0.6	0.84				0.78		
	(0.55–0.66)	(0.79–0.89)				(0.72–0.83)		
AUC **	0.6	0.83						
	(0.55–0.66)	(0.78–0.88)						

**Table 2:** AUC comparison of TIRADS alone versus combined multimodal models. Analysis based on 322 nodules in 208 patients.

	AUC	*p* Value
**ACR-TIRADS (category)**		
TIRADS alone	0.83 (0.78–0.88)	
+ scintigraphy (two-levels)	0.86 (0.81–0.90)	0.039
+ scintigraphy (three-levels)	0.86 (0.81–0.90)	0.039
**ACR-TIRADS (score)**		
TIRADS alone	0.84 (0.79–0.89)	
+ scintigraphy (two-levels)	0.86 (0.81–0.90)	0.064
+ scintigraphy (three-levels)	0.86 (0.81–0.91)	0.063
**EU-TIRADS**		
TIRADS alone	0.78 (0.72–0.83)	
+ scintigraphy (two-levels)	0.80 (0.75–0.85)	0.008
+ scintigraphy (three-levels)	0.80 (0.75–0.85)	0.011

## Data Availability

The data are not publicly available because the informed consent signed by the patients did not provide for it.

## Abbreviations

The following abbreviations are used in this manuscript:

AUC	Area under the receiver operating characteristic curve
ATA	American Thyroid Association
DOR	Diagnostic odds ratio
DLR+	Positive diagnostic likelihood ratio
DLR-	Negative diagnostic likelihood ratio
FNA	Fine-needle aspiration cytology
GEE	Generalized estimating equations
ROC	Receiver operating characteristic
TIRADS	Thyroid Imaging Reporting and Data System
